# Enhanced spatiotemporal resolution imaging of neuronal activity using joint electroencephalography and diffuse optical tomography

**DOI:** 10.1117/1.NPh.8.1.015002

**Published:** 2021-01-01

**Authors:** Jiaming Cao, Theodore J. Huppert, Pulkit Grover, Jana M. Kainerstorfer

**Affiliations:** a Carnegie Mellon University, Department of Biomedical Engineering, Pittsburgh, Pennsylvania, United States; b University of Pittsburgh, Department of Electrical and Computer Engineering Pittsburgh, Pennsylvania, United States; c University of Pittsburgh, Center for Neural Basis of Cognition, Pittsburgh, Pennsylvania, United States; d Carnegie Mellon University, Department of Electrical and Computer Engineering, Pittsburgh, Pennsylvania, United States; e Carnegie Mellon University, Neuroscience Institute, Pittsburgh, Pennsylvania, United States

**Keywords:** electroencephalography, functional near-infrared spectroscopy, diffuse optical tomography, neurovascular coupling, image reconstruction

## Abstract

**Significance:** Electroencephalography (EEG) and functional near-infrared spectroscopy (fNIRS) are both commonly used methodologies for neuronal source reconstruction. While EEG has high temporal resolution (millisecond-scale), its spatial resolution is on the order of centimeters. On the other hand, in comparison to EEG, fNIRS, or diffuse optical tomography (DOT), when used for source reconstruction, can achieve relatively high spatial resolution (millimeter-scale), but its temporal resolution is poor because the hemodynamics that it measures evolve on the order of several seconds. This has important neuroscientific implications: e.g., if two spatially close neuronal sources are activated sequentially with only a small temporal separation, single-modal measurements using either EEG or DOT alone would fail to resolve them correctly.

**Aim:** We attempt to address this issue by performing joint EEG and DOT neuronal source reconstruction.

**Approach:** We propose an algorithm that utilizes DOT reconstruction as the spatial prior of EEG reconstruction, and demonstrate the improvements using simulations based on the ICBM152 brain atlas.

**Results:** We show that neuronal sources can be reconstructed with higher spatiotemporal resolution using our algorithm than using either modality individually. Further, we study how the performance of the proposed algorithm can be affected by the locations of the neuronal sources, and how the performance can be enhanced by improving the placement of EEG electrodes and DOT optodes.

**Conclusions:** We demonstrate using simulations that two sources separated by 2.3-3.3 cm and 50 ms can be recovered accurately using the proposed algorithm by suitably combining EEG and DOT, but not by either in isolation. We also show that the performance can be enhanced by optimizing the electrode and optode placement according to the locations of the neuronal sources.

## 1 Introduction

Electroencephalography (EEG) sensing is widely used for neuronal activity monitoring. Its benefit is the direct measurement of the electrical neuronal activities at high (∼millisecond) temporal resolution. However, the spatial resolution of EEG is low because the distance between the brain and the scalp acts as a spatial low-pass filter.^1^ Additionally, one has to solve a highly ill-posed inverse problem to reconstruct the neuronal sources.^2^ The reconstructed source’s point spread can be unsatisfactorily large (typically on the order of few centimeters),^1^ especially when precise source localization is required, e.g., localizing the seizure focus of epilepsy. Theoretical studies have shown that lower densities of EEG fundamentally limit its spatial resolution.^3^

Functional magnetic resonance imaging (fMRI) is a well-adopted method to measure hemodynamic changes. While the spatial resolution is high (mm-scale) and is far superior to that of EEG, the timescale of hemodynamics is in general on the order of several seconds, making fMRI unable to separate neuronal sources activated with a short temporal separation.

While fMRI is still more commonly used for localizing sources of functional activity, it suffers from drawbacks of being, e.g., not portable, expensive, and not bedside-compatible. Similar to fMRI, functional near-infrared spectroscopy (fNIRS) is a method of measuring hemodynamic changes and can address the above-mentioned issues. When fNIRS is used to reconstruct the neuronal sources, the method is often referred to as diffuse optical tomography (DOT). To reconstruct the source activities using DOT, analogous to EEG, a linear forward model can be calculated and the resulting inverse problem can be solved.^4^ Furthermore, recent work has shown that high spatial precision (mm-scale) is attainable using high-density DOT.^5^ While the linear forward model of DOT also has a spatial low-pass filtering effect as does the EEG forward model, that photons are concentrated in a “banana”-shaped region in tissue near the optical sources and detectors^6^ benefits a potentially higher spatial resolution reconstruction.

Studies in information flow in neural circuits often aim to tease apart dynamics of spatially close neuronal sources that are sequentially activated with only a small temporal separation. Most of the tools for studying these questions strongly rely on understanding precedence:^7^ which computational node got activated (measured by correlation or dependency) earlier in response to a stimulus. For example, the participant taps two different fingers with a separation of 1 s, single-modal measurements using either EEG or hemodynamics recordings (i.e., fMRI or fNIRS/DOT) alone would not suffice to spatiotemporally distinguish between the two neuronal sources. Another example is tracking how the activity evolves is visual processing through different layers of the visual cortex. Here again, the sources are close to each other and are engaged sequentially. In these cases, the centimeter-scale point spread of EEG reconstruction makes the sources spatially indistinguishable to EEG; if they are temporally close (i.e., 1 s or even shorter in the visual example), the slow nature of hemodynamics (∼10  s) would smooth out the responses and make them temporally indistinguishable to fMRI and fNIRS/DOT. Due to the inherently slow nature of the hemodynamic signals (the hemodynamic response lasts a few seconds^8^), even with modern instruments that can sample at as high as 250 Hz,^9^ the temporal resolution of fNIRS/DOT is still fundamentally limited. These fundamental limitations of EEG and hemodynamic recordings motivate the studies of multimodal imaging and joint source reconstruction.

Based on the assumption that evoked neuronal activities and hemodynamic responses are co-localized due to neurovascular coupling,^10^ joint source reconstruction using simultaneous EEG and fMRI has been studied.^2^^,^^11^^–^^13^ In these works, despite the different algorithms used, they largely follow the same idea: the high spatial resolution of fMRI was leveraged to spatially constrain the high temporal resolution EEG reconstruction, and a high spatiotemporal resolution reconstruction of neuronal activities was therefore achieved. It was shown in a work by Liu and He^2^ using simulations that by performing joint source reconstruction, three temporally overlapping neuronal sources that were sequentially activated with a separation of 60 ms were accurately reconstructed, and the spatial content was more confined in comparison to a 128-channel single-modal EEG reconstruction. In the same work, it was also shown *in vivo* that by combining EEG and fMRI, the spatiotemporal dynamics of visual information propagation were accurately reconstructed. Previous works also show that when appropriate algorithms are used (e.g., Twomey, symmetrical methods), combining the two modalities can also help eliminate false detections and false negatives.^12^^,^^14^

Although a plethora of studies have demonstrated the benefits of using EEG and fMRI jointly, given the drawbacks of fMRI, including its high cost and lack of portability, it is of value to study the possibility of using DOT in place of fMRI. While the depth sensitivity of DOT can be limited (<2  cm)^15^ in comparison to fMRI, DOT systems can have much smaller form factors. This is especially promising when considering the recent advancements in instrumenting portable joint EEG-NIRS systems,^16^ which opens up the possibility of wearable multimodal imaging. The limit on how much DOT spatial priors can improve the reconstruction is yet to be understood, but based on the studies that demonstrate DOT reconstruction overlapping accurately with fMRI,^5^ it can be inferred that EEG-DOT has the potential of reaching the performance that is comparable to EEG-fMRI.

Multimodal imaging using simultaneous EEG and fNIRS/DOT has recently been increasingly studied in the fields of neurovascular coupling,^8^ brain-computer interfaces,^17^ and functional activations.^18^ To our knowledge, however, the vast majority of these studies focus on only sensor-level analyses, and very few prior works perform joint reconstruction using EEG and DOT. A method called variational Bayesian multimodal encephalography,^19^ which is based on the variational Bayesian framework,^20^ has previously been proposed by Aihara et al. to incorporate fNIRS as the spatial prior of EEG reconstruction. While Aihara et al. demonstrated, through simulations and experiments, that spatiotemporal reconstruction could be obtained by combining EEG and fNIRS measurements, they did not explicitly examine the important problem of resolving spatiotemporally close sources (e.g., the above-mentioned finger tapping experiment). Another significant limitation in their approach is that, instead of being reconstructed by solving an inverse problem, fNIRS changes were projected onto the cortical surface, which, as we will show in Sec. 3.2, limits the accuracy of the spatial prior. In this paper, we overcome these limitations and propose an algorithm that utilizes DOT reconstruction as the spatial prior of EEG reconstruction using a restricted maximum likelihood (ReML) framework. While high-density DOT can achieve mm-scale resolution,^5^ in this work we specifically target the demonstration of improvement in EEG source localization using more commonly used regular-density DOT systems (i.e., source-detector distance ∼3  cm, no overlapping channels^21^). As will be shown and quantified in Sec. 3, even with a regular-density DOT system, the reconstruction results can be accurate enough to enhance the spatial resolution of EEG. We first show that the algorithm can yield an enhanced spatiotemporal reconstruction of neuronal activities, such that two neuronal sources that are both spatially and temporally close can be clearly distinguished from one another. Further, we show that when fNIRS projection (instead of reconstruction) is used as the spatial prior, the outcome of the same algorithm deteriorates due to the lack of precision of the projection-based spatial prior. We then further examine the limitations of the proposed method under different conditions, such as a variable number of electrodes and sub-optimal optode placement, and discuss how these issues can be addressed.

## 2 Methods

### 2.1 Forward Modeling

#### 2.1.1 Mesh generation

A pre-segmented ICBM152 2009c Nonlinear Asymmetric brain atlas was used,^22^ and for simplicity, the original segmentation was combined into four different tissue types, namely, scalp, skull, cerebrospinal fluid (CSF), and brain. Based on the segmentation, a linear tetrahedral mesh was then created using the iso2mesh toolbox.^23^ This procedure yielded 96,593 nodes and 512,627 tetrahedrons, which is similar to the numbers reported in literature^15^ (see Sec. 4 for further discussion). For both EEG and DOT forward modeling, the source voxels were chosen to be all the mesh nodes that were on the outer surface of the brain compartment, which yielded a total number of 15,255 source locations. Two activation spots each containing 10 voxels (corresponding to a diameter of ∼8  mm, which approximates the spatial spans of digit representations, i.e., mapping of different fingers on the cerebral cortex, in the somatosensory region^24^) in the right hemisphere were activated sequentially with a temporal separation of 50 ms (detailed in Sec. 2.3.1).

#### 2.1.2 EEG forward modeling

**Fig. 1:**
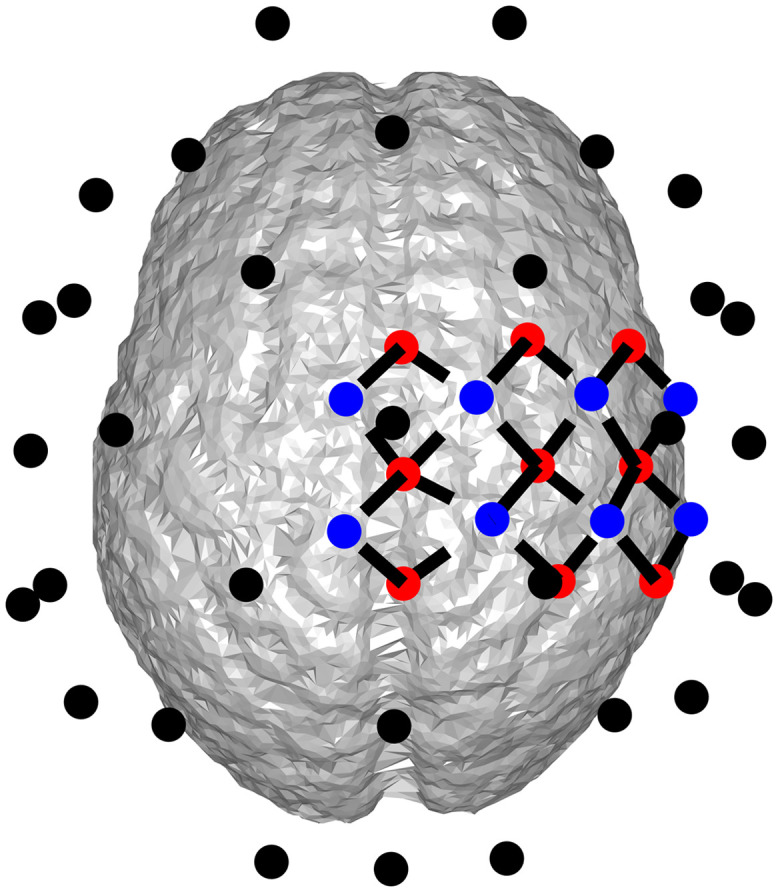
Placement of EEG electrodes and and DOT probes. Black dots: EEG electrodes; blue dots: DOT detectors; red dots: DOT sources; dashed lines: DOT channels formed by source-detector pairs.

The electrodes were placed according to the standard 32-channel (also 64-channel in one example) 10–20 system, using the template provided in the Fieldtrip toolbox.^25^ The locations of the electrodes are shown in Fig. 1. The resistances of the four layers were assumed to have a ratio of (from scalp to brain) 1:80:1/5:1 according to literature.^26^ The leadfield matrix was then calculated in Fieldtrip using the SimBio toolbox.^27^^,^^28^ Finally, at each dipole location, the 3D leadfield vector given by Fieldtrip was projected onto the direction normal to the cortical surface.

#### 2.1.3 DOT forward modeling

Optical sources and detectors were sparsely placed over the right motor and somatosensory cortex of the brain. The configuration of the optodes is shown in Fig. 1, totaling 24 channels. Within each channel, the separation between the optical source and the optical detector ranged from 1.7 to 2.7 cm. Wavelengths of the system (namely, 750 and 850 nm) and the corresponding optical properties of different tissues were chosen according to literature.^5^ Finally, the forward matrix of DOT was calculated using the NIRFAST toolbox,^4^ during which procedure only two chromophores, oxygenated hemoglobin (HbO) and deoxygenated hemoglobin (Hb) were considered. The forward model calculates optical density changes (ΔOD) using the changes of HbO and Hb concentrations (ΔHbO and ΔHb, respectively).

### 2.2 Inverse Problem Using Restricted Maximum Likelihood Method

The inverse problems (EEG only, DOT only, and joint, detailed in Sec. 2.3.2) were all solved using an ReML algorithm.^29^ If the forward problem is defined in the linear form,^29^

Y=Hβ+ϵ,

where Y is the measurement vector, H is the forward matrix, β is the neuronal source vector, and ϵ is the sensor noise, the algorithm attempts to solve the following optimization problem:

$$\arg\;\max_{\beta ,C_{N},C_{P}}{\left\|Y-H\beta \right\|}_{C_{N}^{-1}}^{2}-{\left\|\beta \right\|}_{C_{P}^{-1}}^{2}-\log|C_{N}|-\log|C_{P}|,$$

where $C_{N}$ denotes the covariance matrix of measurement noise, $C_{P}$ denotes the covariance matrix of the prior distribution of the neuronal sources, and for some arbitrary matrices X and M, the notation ${\left\|X\right\|}_{M}$ denotes the weighted norm: ${\left\|X\right\|}_{M}^{2}=X^{T}\mathrm{MX}$. Such formulation is based on the assumption that both the neuronal sources and the sensor noise follow zero-mean normal distributions, and is derived from the maximum a posteriori estimation. It is worth noting that when $C_{N}$ and $C_{P}$ are both diagonal matrices with equal diagonal elements, the problem reduces to the commonly used Tikhonov regularization.^29^ In EEG, β represents the electrical activities, Y indicates the scalp EEG recordings, and H is the leadfield matrix calculated using Fieldtrip.^25^ In DOT, β represents ΔHbO and ΔHb, Y represents ΔOD, and H is the Jacobian calculated using NIRFAST.^4^^,^^29^

When using ReML, instead of solving directly for $C_{N}$ and $C_{P}$, structural assumptions can be made on the covariance matrices by rewriting them in forms of linear decomposition, i.e.,

$$C_{N}=\underset{i}{\sum }\Lambda_{N,i}Q_{N,i},\;C_{P}=\underset{i}{\sum }\Lambda_{P,i}Q_{P,i},$$

where $Q_{N,i}$ and $Q_{P,i}$ are the symmetric matrices representing the components to construct the covariance matrices, and $\Lambda_{N,i}$ and $\Lambda_{P,i}$ are the coefficients to be estimated from the data. Such decomposition provides one with greater flexibility when making assumptions on the covariances, e.g. different wavelengths in DOT may have different measurement noises, source voxels in different brain regions may have different levels of activities.

### 2.3 Simulated Experiments

#### 2.3.1 Data simulation

**Fig. 2:**
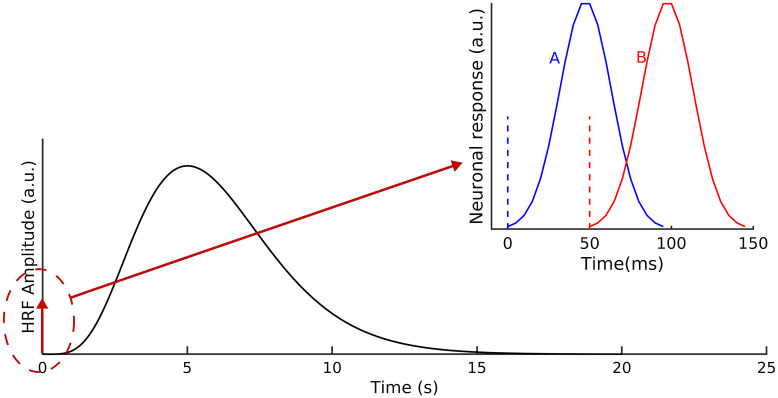
The main figure shows the HRF in response to a single stimulation trial. The stimulation is marked as the red arrow at time 0. A zoomed-in version can be found in the small figure, where the two individual stimuli are shown. Also shown in the small figure are the neuronal electrical responses to the individual stimuli.

A block of 30 s was simulated, with the first 20 s containing 100 repeated trials separated by 200 ms. Within each trial, two stimuli were 50 ms apart from each other, each evoking a response at one of the activation spots, as is shown in the top right subfigure of Fig. 2.

In demonstration of the limitations of using single-modal reconstruction and the improvement using the proposed method, the activation spots were chosen to be spatially close (2.3 to 3.3 cm, in the different cases simulated), such that EEG alone would not accurately distinguish between the two spatial locations. On the other hand, the 50-ms temporal separation was chosen such that the temporal information could not be resolved using the slow hemodynamics obtained by DOT. We also altered the spatial locations of the two activation spots, such that the effectiveness and limitations of the proposed algorithm were also tested.

To simulate the source level activities, we made the assumption that electrical and hemodynamic activities are co-localized. In particular, for each brain voxel, we represented the neuronal responses using a train of impulse functions, where each impulse corresponded to an activation event. After that, the same train of impulse functions was convolved with electrical and hemodynamic response functions (HRFs), respectively, to simulate the source level activations. This assumed structure (i.e., same activation being convolved with respective response functions) is analogous to that used in a work by Valdes-Sosa.^30^

In response to each functional stimulus, the neuronal response was assumed to follow a simple bell shape in time,^2^ as is shown in Fig. 2. In addition, an independent and identically distributed (i.i.d.) Gaussian noise was added to all source voxels at each time point to represent the background activities. The background activities were assumed to have a standard deviation of 1/20 of the maximum amplitude of evoked neuronal activities, which is slightly higher than the 1/50 that is used in literature.^19^

Meanwhile, HbO and Hb were assumed to increase and decrease (respectively) following a canonical HRF,^31^ the shape of which is shown in Fig. 2. ΔHbO and ΔHb were assumed to be out of phase (i.e., 180° phase difference), and the ratio of the maximum amplitudes was set to 3:1, similar to values found in literature.^29^ Analogous to EEG, i.i.d. Gaussian background activities were added to each source voxel at each time point to better reflect reality. The standard deviation of background activities added to ΔHbO was chosen to be four times of the maximum amplitude, and for ΔHb, the background activities were assumed to have a standard deviation of two times of the maximum amplitude. These values are estimated from a public fNIRS dataset.^17^

The simulated brain activities were then mapped to EEG and DOT sensor-space recordings using the above-mentioned forward models. Additive Gaussian noises were added to both EEG and DOT recordings, and the signal-to-noise ratios (SNRs, defined as the square amplitude of signal divided by the variance of noise) were chosen to be 10:1, 10:1, and 20:1 for EEG, 750-nm laser, and 850-nm laser, respectively, which are consistent with literature.^29^^,^^32^

#### 2.3.2 Joint reconstruction using EEG and DOT

Prior to reconstruction, a trial average was performed on EEG, i.e., the reconstruction algorithm was run on only one averaged trial of EEG. For DOT, the signals were first low-pass filtered under 1 Hz using a second order Butterworth filter to reduce noise, and since the hemodynamic response is not fast enough to have trial-level resolution, the average ΔOD over the entire block was taken.

To demonstrate the improvement using the proposed method, we ran the following simulations:

EEG only: We first performed source reconstruction using only EEG, and demonstrated the limitations in spatial resolution. ReML was applied to each time step of the averaged trial individually, and at each time step, both sensor noise and brain activities were assumed to be i.i.d. The covariance matrices can therefore be written as

$$C_{N}=\Lambda_{N}I_{N},\;C_{P}=\Lambda_{P}I_{P},$$

where $I_{N}$ and $I_{P}$ are identity matrices with appropriate sizes.

DOT only: Next, we showed that despite the lack of temporal information, DOT reconstruction could achieve much higher spatial resolution than EEG, which was quantified by the “bias-spread metric”(BSM) (see Sec. 2.3.3). ReML was applied to the block-averaged ΔOD. To construct the covariance matrices, we assumed that within each wavelength, the sensor noise was i.i.d. within each wavelength, but the variances were different at the wavelengths. Similarly, we assumed that ΔHbO and ΔHb were also both i.i.d. with different variances. This yields the following decomposition of $C_{N}$ and $C_{P}$,

$$C_{N}=\Lambda_{N,1}(\begin{array}{cc} I & 0\\ 0 & 0 \end{array})+\Lambda_{N,2}(\begin{array}{cc} 0 & 0\\ 0 & I \end{array}),\;C_{P}=\Lambda_{P,1}(\begin{array}{cc} I & 0\\ 0 & 0 \end{array})+\Lambda_{P,2}(\begin{array}{cc} 0 & 0\\ 0 & I \end{array}),$$

where 0 and I represent zero and identity matrices with appropriate sizes, respectively.

DOT as EEG spatial prior: Finally, we used the results of ΔHbO reconstruction from DOT as the spatial prior of EEG reconstruction, and showed that by doing so, high spatiotemporal resolution could be achieved. It is worthy to note that theoretically, either HbO or Hb can be used to construct the spatial prior, however, in practice, it is generally more favorable to use HbO due to the typically higher SNR.^29^ Intuitively, when constructing $C_{P}$ for EEG, we still assume the sources to be independent, but a voxel should have larger variance if it shows high activity in DOT. If we define

β′=|β^HbO|∘1{|β^HbO|>k}max|β^HbO|,

where ${\hat{\beta }}_{HbO}$ denotes the reconstructed HbO activities, ∘ denotes Hadamard (element-wise) product, k is a threshold set to eliminate the influence of small amplitude artifacts, and 1{X} is an indicator function whose output is the same size as X, and an element is 1 if the corresponding element in X is true and 0 otherwise. The diagonal elements in $Q_{P}$ can then be defined as

$$Q_{P\{i,i\}}=1-\exp(-\frac{\beta_{i}^{\prime }+a}{b}),$$

where the subscript i denotes the i’th element. Such function is chosen to control how the EEG reconstruction depends on the DOT prior. Specifically, parameter a determines the baseline activity allowed for each voxel (the smaller it is, the more biased is the reconstruction toward the DOT prior, but it also more likely to cause numerical issues due to the overly strict constraints), and b determines the “dynamic range,” i.e., given the same HbO activities, what is the ratio between the largest and smallest values in the resulting EEG variances (the smaller b is, the larger the dynamic range, but also the more likely it is to cause spiky results). In the results shown below, unless otherwise stated, we chose k=0.1, a=0.1, and b=1. In particular, k was chosen empirically to filter out the low-amplitude ripples in the DOT reconstruction, and as can be seen in the Appendix, as long as a and b are within a certain range [roughly, a∈(0.05,0.1), b∈(1,5)], their impact on the improvement of spatiotemporal accuracy is small. The values a=0.1 and b=1 were therefore somewhat heuristically chosen for both good performance and numerical stability.

#### 2.3.3 Metrics

To quantitatively measure the goodness of a reconstruction, we propose to use a BSM, which is analogous to mean square error. To calculate the metric, the reconstruction result is first thresholded, such that at each time step, all voxels with amplitudes of at least half of maximum are considered as “valid” voxels, which corresponds to the commonly used FWHM metric in the DOT field.^21^

After thresholding, the bias term is defined as the Euclidean distance between the center of mass of the valid voxels and the true center. The spread term is defined as the mean-square distance between all valid voxels to the center of mass of the valid voxels, i.e.,

$$spread=\frac{1}{N(\{valid\})}\underset{i\in \{valid\}}{\sum }{\left\|r_{i}-r_{c}\right\|}_{2}^{2},$$

where N() denotes the size of a set, $r_{i}$ is the location vector of the i’th voxel, and $r_{c}$ is the location vector of the center of mass of the valid voxels.

When two spots are simultaneously activated, bias and spread are calculated separately for each of them. In the case where only one region is reconstructed (e.g., two activation spots are indistinguishable in the reconstruction, or one region is missing), if all valid voxels are closer to one activation spot (say, A) than the other (say, B), we report that activation spot B is not detected. Otherwise, the one reconstructed activation is used to calculate bias and spread for both A and B.

Finally, the BSM is defined as

$$BSM=\sqrt{bias^{2}+spread},$$

and has the units of distance.

## 3 Results

The algorithm was implemented using Matlab (Mathworks Inc., Massachusetts), and the ReML part was adapted from the implementation in the NIRS Brain AnalyzIR toolbox.^33^ The codes for generating the forward models and simulating the results are available at https://github.com/JiamingCao/NIRS-EEG. All reconstruction results shown were normalized to the maximum value of each individual reconstruction.

### 3.1 Joint Source Reconstruction Using EEG and DOT can Achieve High Spatial-Temporal Resolution

**Fig. 3:**
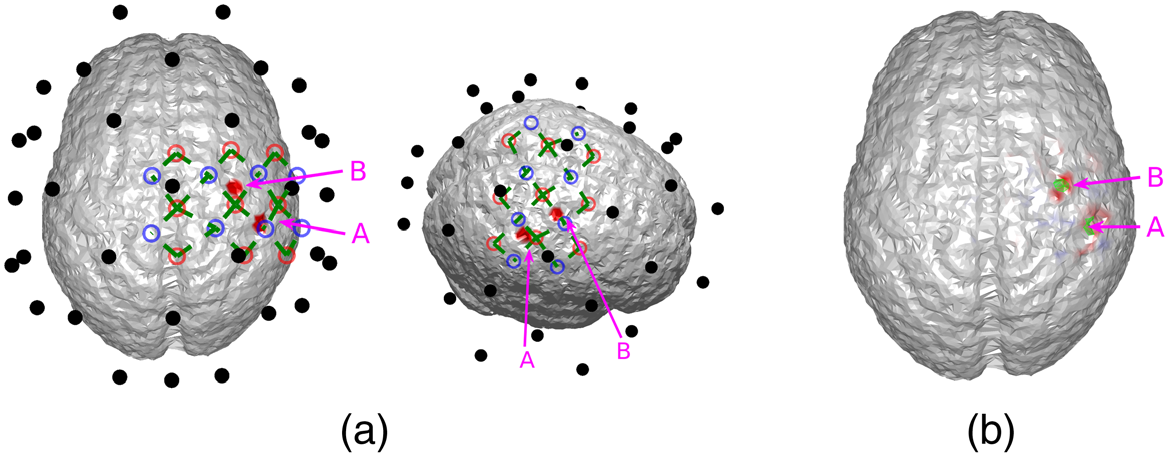
(a) Locations of the activation spots and their relative position to EEG and DOT channels. Neither of them are close to EEG electrodes. However, they are both well-covered by the DOT grid. The same locations are shown in different angles for better illustration. (b) Reconstructed locations of the activation spots using DOT, showing that the location of HbO increase corresponds to the ground truth locations. The overlaid green regions indicate the true neuronal activation.

We first demonstrate that joint neuronal source reconstruction using DOT as the spatial prior of EEG reconstruction can resolve spatiotemporally close neuronal sources. For this purpose, we have simulated a case where neither of the activation spots are close to the EEG electrodes, such that spatially, EEG reconstructs the two activation spots with poor accuracy, but both locations can be resolved using DOT reconstruction. The locations of the activation spots and the DOT-reconstructed locations are shown in Fig. 3. In this case, the two activation spots are separated by ∼2.4  cm.

**Fig. 4:**
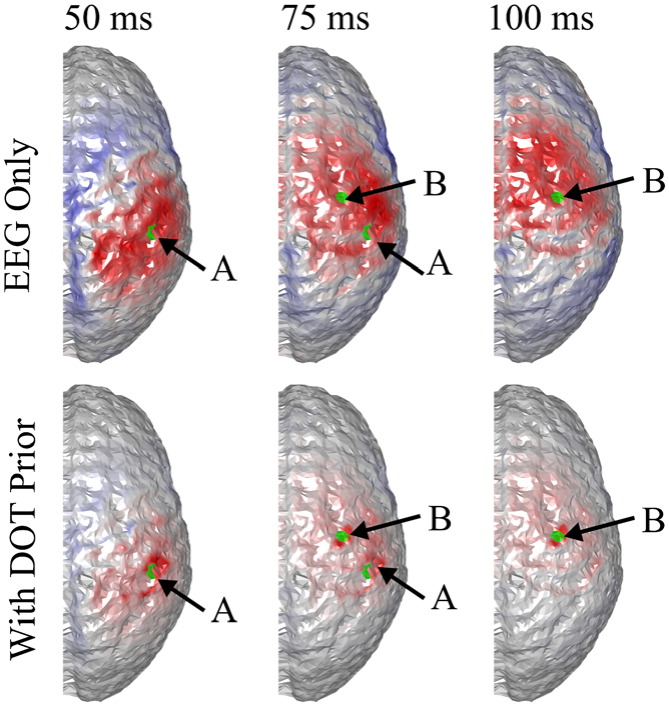
Comparison of true neuronal activation, reconstruction using only EEG, and reconstruction of EEG using DOT prior at different times. The overlaid green regions indicate the true neuronal activation. Reconstruction using only EEG has low spatial resolution, but when utilizing the DOT-based prior, the results present more of the characteristics of the true neuronal activation pattern. Only the right hemisphere is shown, as no significant activation is observed on the left hemisphere.

It can be seen that the reconstructed locations of the activation spots using DOT agree very well with the locations of the true neuronal activation, which can be quantified using BSM: at spots A and B, the BSM values are 5.7 and 5 mm, respectively. Results of reconstruction using only EEG, and reconstruction of EEG using DOT as spatial prior are shown in Fig. 4. Different time points are shown, namely 50, 75, and 100 ms, where the neuronal response of region A peaks, responses of regions A and B have equal amplitudes, and response of region B peaks, respectively (see Fig. 2). Also shown in Fig. 4 are the true neuronal activations at these time points, as illustrated by the overlaying green patches. Using only EEG for reconstruction yields a large spread as seen in Fig. 4, first row. When using DOT as the spatial prior (Fig. 4 second row), the reconstruction is more confined and shows the expected spatiotemporal characteristics of the true neuronal activation. These improvements of DOT-based reconstruction in EEG are shown quantitatively in Table 1, using the bias-spread metric.

**Table 1:** Quantitative comparison of reconstruction using only EEG, EEG with DOT reconstruction prior, and EEG with fNIRS projection prior in the case shown in Figs. 4 and 5. The BSM values suggest that both DOT reconstruction prior and fNIRS projection prior can improve the spatiotemporal resolution of neuronal source reconstruction, but the enhancement using the latter is limited.

	50 ms, spot A	75 ms, spot A	75 ms, spot B	100 ms, spot B
EEG only	22.3 mm	32.6 mm	26.1 mm	29.5 mm
With reconstruction prior	12.8 mm	11.1 mm	8.4 mm	4.9 mm
With projection prior	14.2 mm	27.5 mm	18.7 mm	16.3 mm

### 3.2 Using fNIRS Projection Prior Limits the Improvement of Reconstruction Accuracy

We further compared EEG reconstruction using DOT-based prior, which provides a 3D reconstruction of hemodynamic changes, to that using a simpler projection prior as Aihara et al.^19^ work has done. For this, we used the same activation spots and sensor configuration shown in Figs. 3 and 4.

**Fig. 5:**
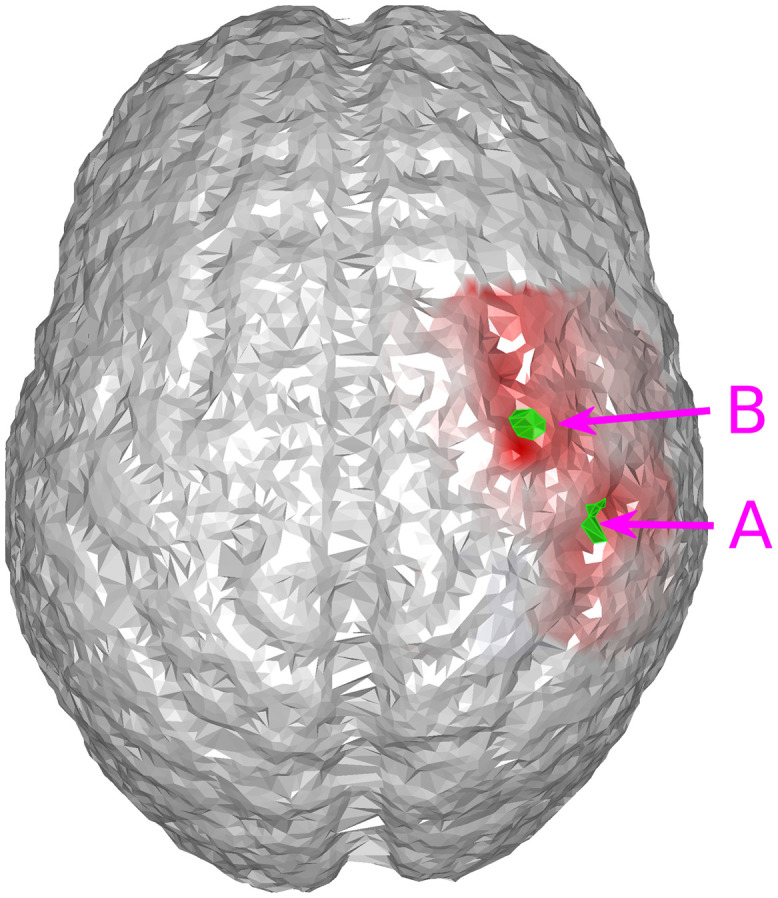
HbO activities projected onto the cortical surface. The activation spots are the same as in Fig. 3(b), but they are not resolved as clearly. The overlaid green regions indicate the true neuronal activation.

Figure 5 shows the results of cortical projection of HbO activities. The sensor-space HbO activities were calculated using the modified Beer–Lambert law,^34^ projected onto the cortical surface using the convex hull method,^35^ and then interpolated on the cortical surface.^19^ In comparison to DOT [Fig. 3(b)], the projection prior has much larger spread. Quantitatively, the BSM of the projection prior at spots A and B are 26.3 and 22.7 mm, which are much larger than those of the reconstruction prior.

**Fig. 6:**
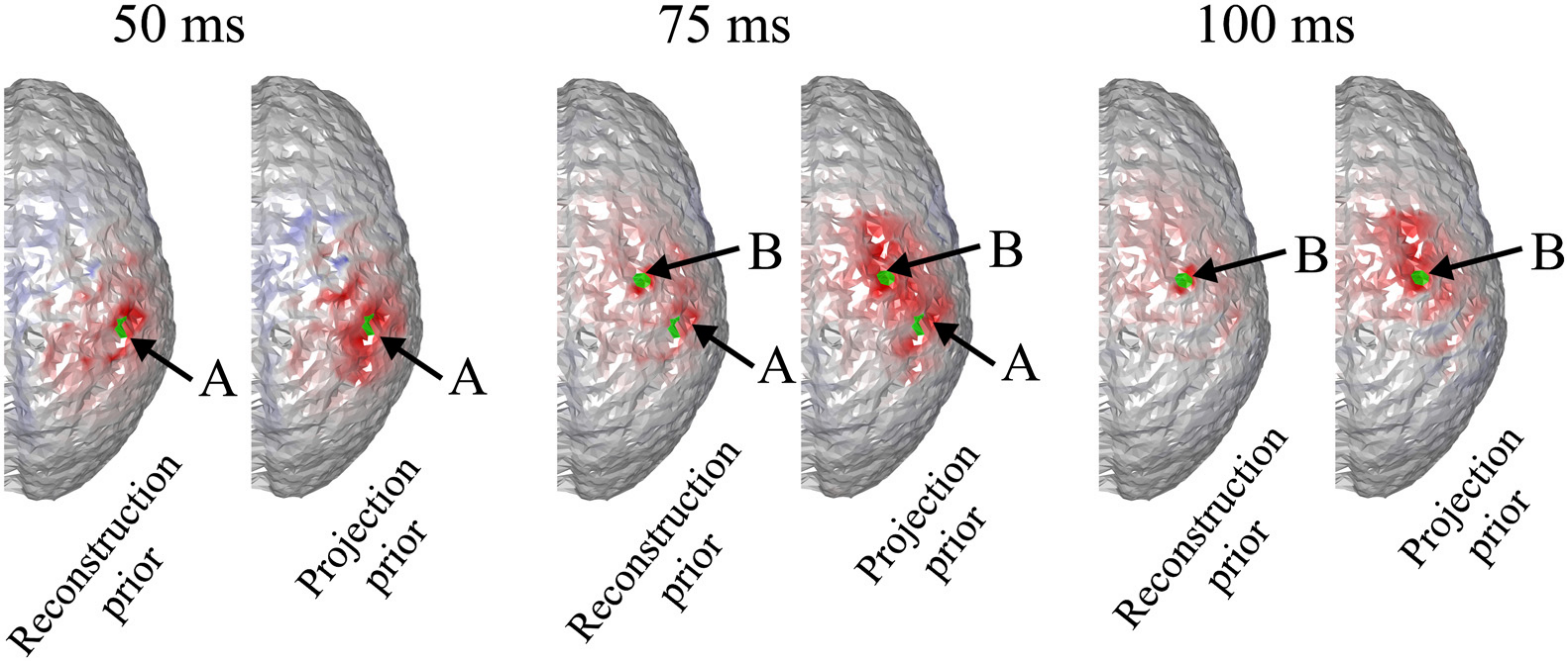
Comparison of true neuronal activation, EEG reconstruction using reconstructed HbO as prior, and EEG reconstruction using projected HbO as prior, at different timesteps. The overlaid green regions indicate the true neuronal activation. Shown is the same case as in Fig. 4. It can be seen that while the two activation spots are correctly resolved, the results using projected HbO as prior show less spatial precision.

Consequently, when the projection-based spatial prior is used for EEG reconstruction, the spatial resolution is not substantially enhanced compared to using a DOT-based prior. A comparison of EEG reconstruction using DOT-based prior and projection-based prior at different time points is shown in Fig. 6. The comparison of reconstruction accuracy in EEG is quantified in Table 1 in terms of BSM.

### 3.3 Number of EEG Electrodes can Limit the Accuracy of Reconstruction

**Fig. 7:**
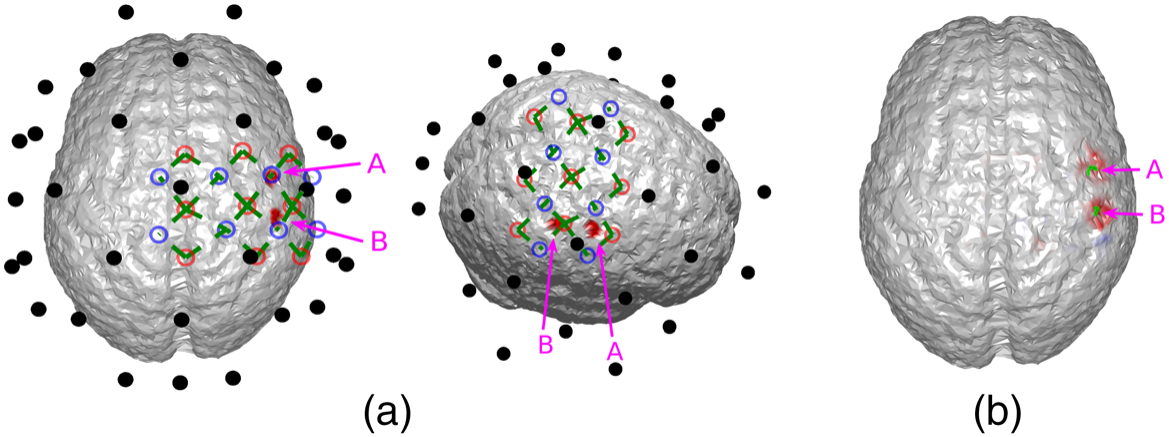
(a) Locations of the activation spots and their relative position to EEG and DOT channels. They are both very close to one EEG electrode. The same locations are shown in different angles for better illustration. (b) Reconstructed locations of the activation spots using DOT. Despite that they are both close to the same EEG electrode, they are very well resolved using DOT. The overlaid green regions indicate the true neuronal activation.

**Fig. 8:**
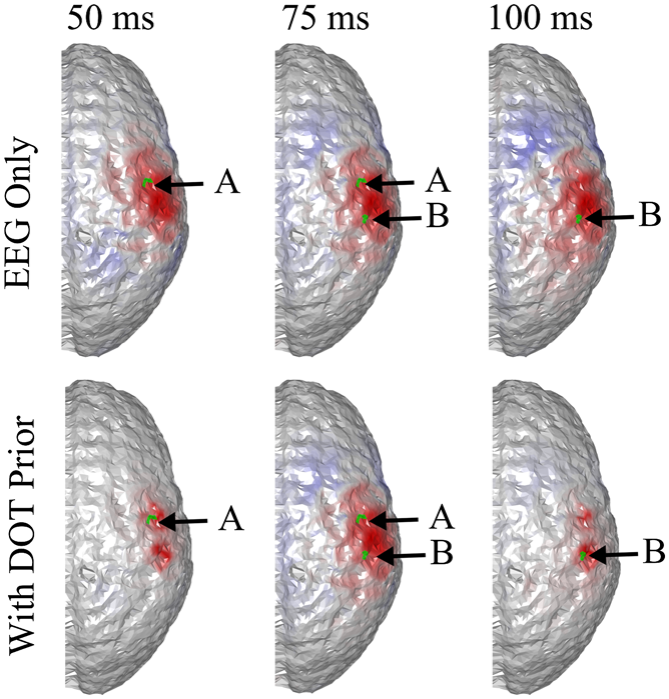
Comparison of true neuronal activation, reconstruction using only EEG, and reconstruction of EEG using DOT prior at different times. The overlaid green regions indicate the true neuronal activation. Since both activation spots are close to the same EEG electrodes, they are hardly distinguishable in construction using only EEG. In this case, improvement using DOT as spatial prior is minimal. Only the right hemisphere is shown, as no significant activation is observed on the left hemisphere.

To explore the limitations of the proposed method, we simulated different scenarios where either the sensitivity of EEG or the the accuracy of DOT was unsatisfactory. First, we simulated the case of insufficient EEG sensitivity, where two activation spots were placed close to the same EEG electrode [Fig. 7(a)]. In this case, the two activation spots are ∼2.3  cm apart. The BSM values for activation spots A and B of the DOT prior are 8.9 and 6.2 mm, suggesting an accurate spatial prior. It can be seen that the two locations are hardly distinguishable in the reconstruction of 32-channel EEG (first row in Fig. 8), since the signals from both activation spots are almost equally picked up by just one electrode. As is seen in Fig. 7(b), the two activation spots are not spatiotemporally differentiated, even though DOT provides a high spatial resolution prior. This suggests that better inference from EEG is needed. The localization accuracy is quantified using BSM in Table 2. To overcome this issue, a higher density EEG system can be used.

**Table 2:** Quantitative comparison of reconstruction using 32-channel EEG, 64-channel EEG, and the former two with DOT prior, in the case shown in Fig. 8 and Fig. 9. The BSM values suggest that although without spatial prior using more EEG electrodes contributes to only small improvement, the extra information of the DOT-based prior can lead to a more substantial improvement of reconstruction accuracy.

	50 ms, spot A	75 ms, spot A	75 ms, spot B	100 ms, spot B
32-channel EEG only	16.7 mm	18.7 mm	18.3 mm	17.0 mm
64-channel EEG only	14.3 mm	18.0 mm	15.7 mm	17.0 mm
32-channel EEG with prior	16.7 mm	9.1 mm	7.6 mm	11.3 mm
64-channel EEG with prior	14.4 mm	9.0 mm	7.8 mm	9.9 mm

#### 3.3.1 Improvement using a higher density EEG

**Fig. 9:**
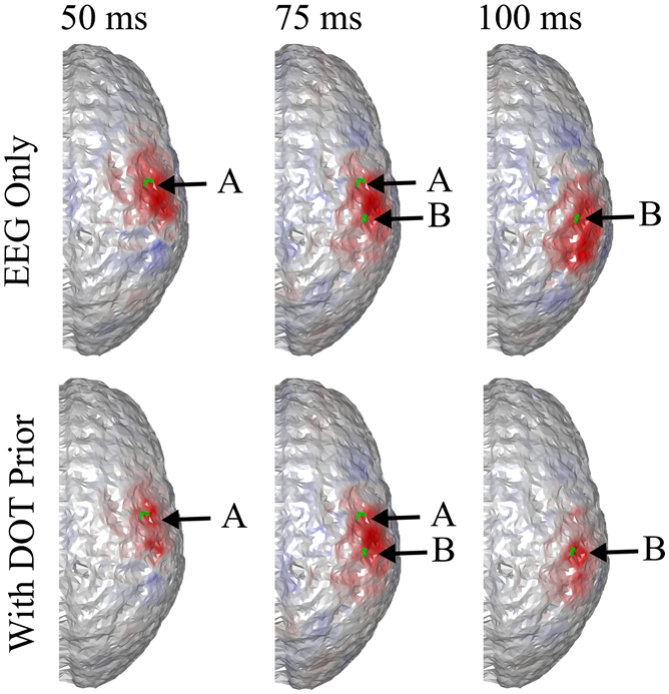
Activation spots are the same as in Fig. 8, but 64 EEG channels were used. Spots A and B become distinguishable in the denser grid, and DOT prior sharpens the reconstruction, especially that of B. Only the right hemisphere is shown, as no significant activation is observed on the left hemisphere. The overlaid green regions indicate the true neuronal activation.

As is shown in Fig. 9, when the number of electrodes is increased to 64 and the same algorithm is applied (with a chosen to be 0.25), spots A and B are distinguishable using EEG, and DOT prior helps improve the spatial precision, allowing the two activation spots to be spatiotemporally resolved. The improvement is shown quantitatively in Table 2.

### 3.4 Sub-Optimal Placement of Optodes can Deteriorate the Accuracy of Reconstruction

**Fig. 10:**
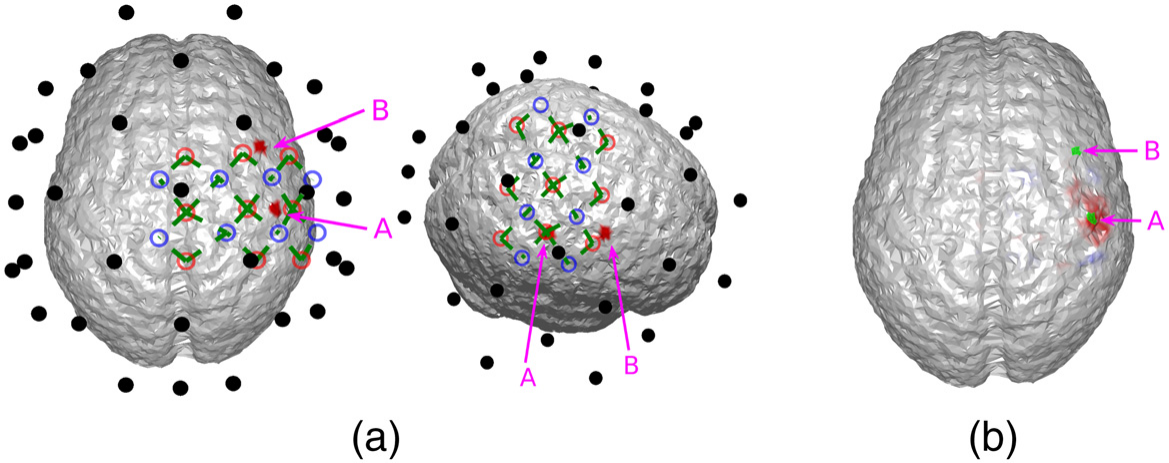
(a) Locations of the activation spots and their relative position to EEG and DOT channels. They are each close a separate EEG electrode, leading to EEG distinguishability, but only one of them is detectable by the DOT grid. The same locations are shown in different angles for better illustration. (b) Reconstructed locations of the activation spots using DOT. Only spot A can be seen in the reconstruction. The overlaid green regions indicate the true neuronal activation.

**Fig. 11:**
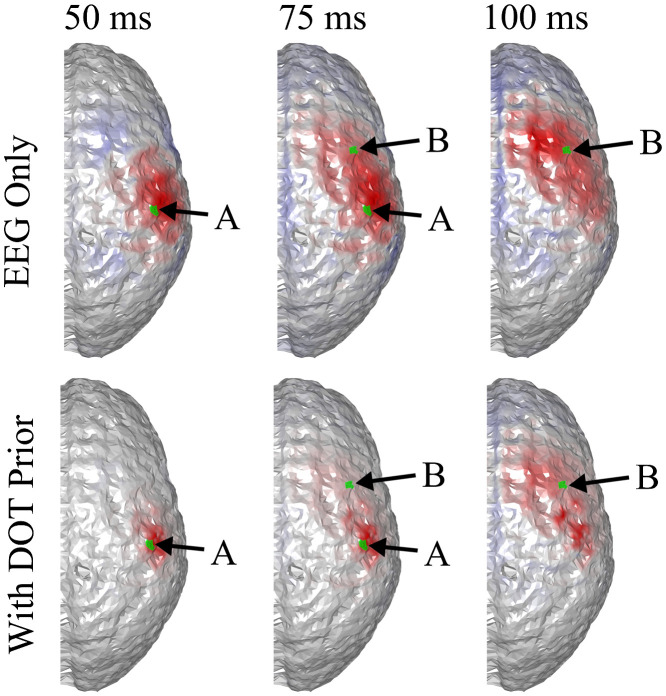
Comparison of true neuronal activation, reconstruction using only EEG, and reconstruction of EEG using DOT prior at different times. The overlaid green regions indicate the true neuronal activation. Despite a lack of precision, EEG reconstruction can distinguish between the two activation spots. However, when spot B is activated, DOT acts as an incorrect spatial prior because the prior shows only the location of spot A, such that the results are worsened. Only the right hemisphere is shown, as no significant activation is observed on the left hemisphere.

Finally, we show a case where DOT provides an incorrect prior to EEG reconstruction, and worsens the results. Specifically, we chose the location of activation spot B to be outside of the DOT grid, reducing the sensitivity of DOT to it. In this case, the two activation spots were each close to a separate EEG channel, implying a high EEG detectability, but only spot A was detected by the DOT grid, as is shown in Fig. 10. The distance between the two activation spots is ∼3.3  cm, and when calculating the BSM, the value is 6.8 mm for spot A, and spot B is reported to be undetected. Using this prior, although the DOT prior makes the reconstruction of spot A more confined, when spot B was activated, DOT in fact provided an incorrect prior. Consequently, as can be seen in the lower right subfigure of Fig. 11, the reconstruction is biased toward spot A, making the results worse then using only EEG. This can also be seen in Table 3 in terms of BSM values. To address this issue, a higher density DOT grid, or optimized optode placement according to the regions of interest can be used.

**Table 3:** Quantitative comparison of reconstruction using only EEG, EEG with incorrect DOT prior, and EEG with enhanced prior, in the case shown in Fig. 11 and Fig. 13. The BSM values clearly suggest that when an incorrect prior is used, the accuracy of reconstruction can be exceptionally bad. Using a correct prior, however, can substantially improve the reconstruction results.

	50 ms, spot A	75 ms, spot A	75 ms, spot B	100 ms, spot B
EEG only	14.7 mm	19.8 mm	27.6 mm	17.5 mm
With incorrect DOT prior	7.7 mm	8.8 mm	15.2 mm	24.2 mm
With improved DOT prior	7.1 mm	8.2 mm	8.3 mm	6.3 mm

#### 3.4.1 Improvement using an additional optical detector

**Fig. 12:**
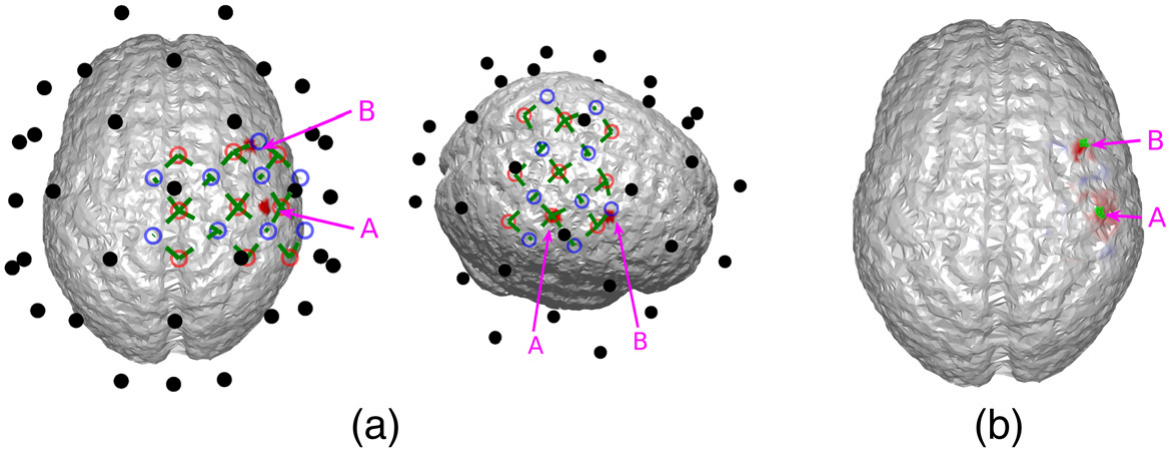
(a) Locations of the activation spots are the same as in Fig. 10(a), but an additional optical detector was used (top right), leading to two extra channels, and hence better coverage of the cortex. The same locations are shown in different angles for better illustration. (b) Using the augmented DOT grid, both spots A and B can be reconstructed. The overlaid green regions indicate the true neuronal activation.

**Fig. 13:**
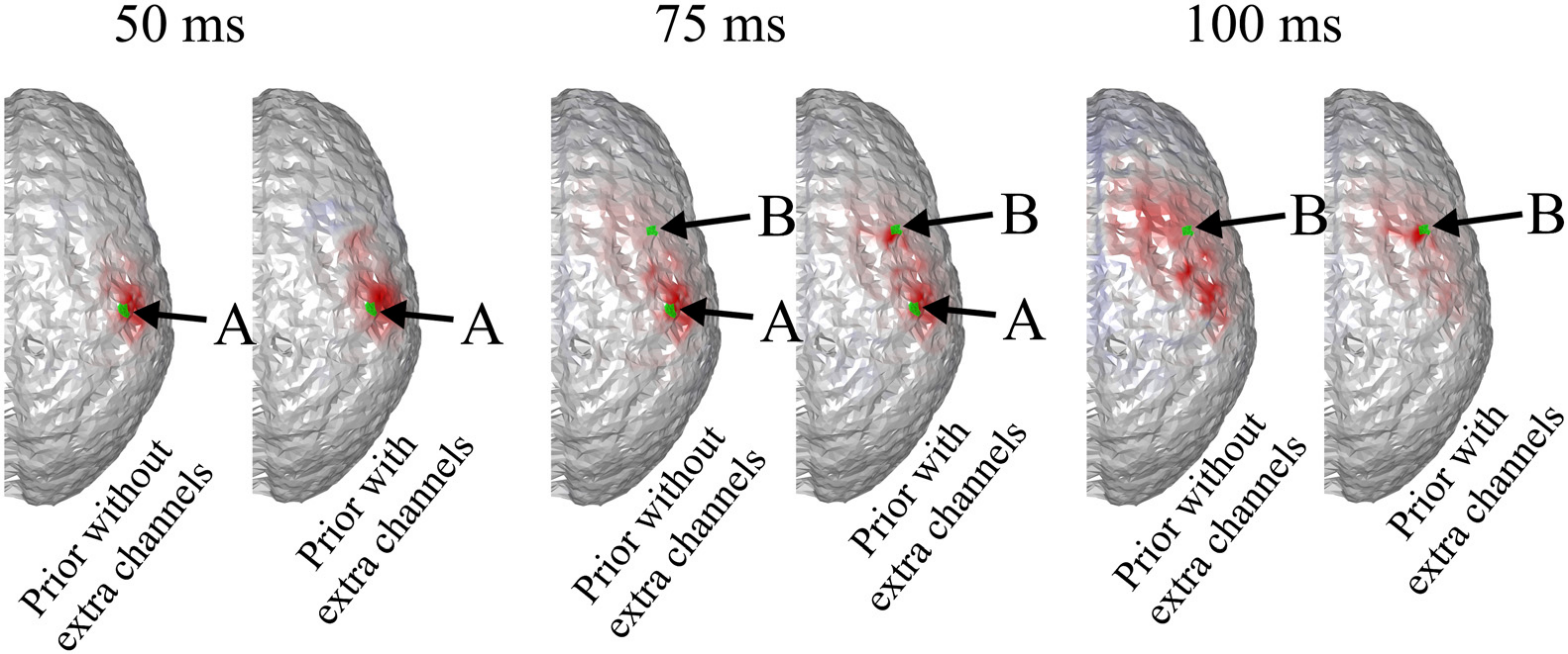
Comparison of EEG reconstruction in Fig. 11 and EEG reconstruction using prior reconstructed with the extra channels. Locations of the activation spots are the same as in Fig. 10(a). When both spots A and B are reconstructed using DOT, as is shown in Fig. 12, DOT prior can enhance the spatial precision of EEG reconstruction. Only the right hemisphere is shown, as no significant activation is observed on the left hemisphere. The overlaid green regions indicate the true neuronal activation.

To show that improving the placement of DOT optodes can address the issues caused by incorrect DOT priors, we added an extra optical detector in the grid, which contributed to two extra channels [Fig. 12(a)]. Such an augmented grid allows the previously invisible activation spot B to also appear in the reconstruction [Fig. 12(b)], leading to a “correct” spatial prior. As a result, as is shown in Fig. 13 and Table 3, compared to the results shown in Fig. 11, the spatial accuracy of EEG reconstruction using the improved spatial prior is substantially enhanced.

### 3.5 Systematic Assessment of the Effectiveness of the Algorithm

**Fig. 14:**
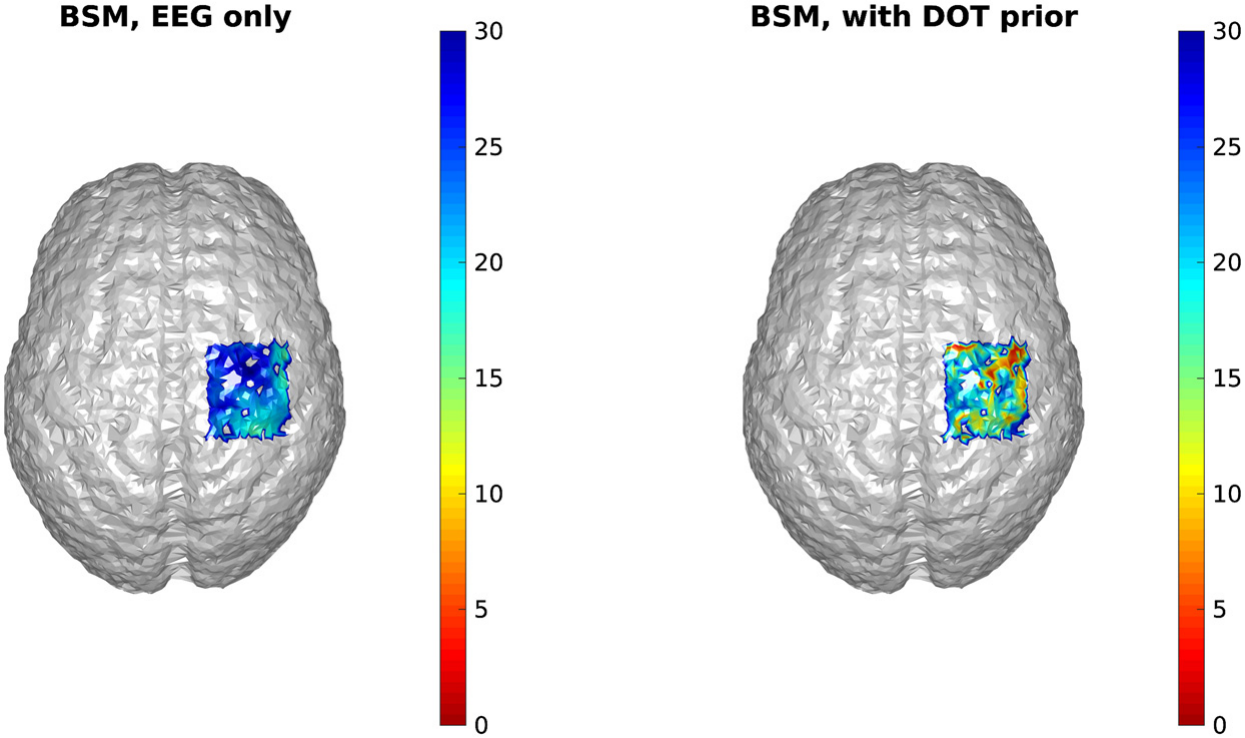
Comparison of BSM values using only EEG and EEG with DOT prior. Shown are 1000 simulated locations, and an overall improvement of reconstruction accuracy as measured by BSM can be observed. The colorbars have the units of mm.

To systematically assess the effectiveness of the proposed algorithm, we first tested the improvement of only spatial accuracy by simulating 1000 randomly chosen activation locations that were approximately in the right sensory-motor cortex, which is the same region that was simulated in the previous sections and is well-covered by the DOT grid. The BSM values of using only EEG and using EEG with DOT prior are shown in Fig. 14. At 83.4% of the locations, DOT led to an improvement of spatial accuracy, which can also be observed from the color map in Fig. 14. When using EEG only, the BSM values of the 1000 locations averaged at 22.97 mm with a standard deviation of 3.48 mm. In comparison, when the DOT prior was used, the average BSM became 16.22 mm, and the standard deviation became 6.32 mm. We statistically tested whether the use of DOT prior led to a decrease in average BSM using a single-sided two-sample t-test, and we observed an extremely small p-value ($5.3\times 10^{-153}$).

Further, we performed spatiotemporal reconstructions of 500 randomly chosen combinations of activation spots. Within each combination, analogous to the cases shown above, two activation spots were used. They were chosen to be both approximately in the same sensory-motor region, and had a spatial separation of 2 to 3 cm. Analogous to the single-location case previously mentioned, we calculated the average BSM of spots A and B with and without DOT prior. Interestingly, we did observe that in certain cases, one of the spots were deemed “undetected,” suggesting the difficulty of resolving two simultaneously activated brain regions. When only EEG was used for reconstruction, in 487 out of 500 (97.4%) simulated combinations, both spots were detected. Among them, the averaged BSM values of the two spots were 28.76±5.92  mm (standard deviation) and 24.59±4.58  mm, respectively. When DOT prior was used, the combinations where both spots were detected became 422 of the 500 (84.4%), and among them, the averaged BSM values were calculated to be 16.26±8.39  mm and 16.71±7.69  mm, respectively. We statistically tested whether the use of DOT led to a decrease in average BSM for both spots using single-sided two-sample t-tests, and the p-values were again observed to be extremely small ($2.1\times 10^{-106}$ and $9.8\times 10^{-62}$, respectively). It is worhy to note that the number of combinations where both spots were detected was lower when DOT prior was used. This suggests that a regular-density, unoptimized DOT grid may occasionally provide incorrect priors that worsen the reconstruction results, as was discussed in Sec. 3.4. However, when the priors are correct, the improvement can be significant. Finally, we assess the improvement of spatial-temporal resolution by examining the BSM values of the two spots at different time steps. In particular, for each simulation, we examine the improvement of BSM values of spot A at 50 ms, spot A at 75 ms, spot B at 75 ms, and spot B at 100 ms. We say that we see an improvement in a simulation only when BSM improvements (i.e., smaller values) are seen in all the four comparisons. In our results, 258 of the 500 simulations demonstrated improvement, which corresponds to a percentage of 51.6%.

The percentage of improved spatiotemporal reconstructions (51.6%) is lower in comparison to spatial-only reconstruction (85.4%), due to the extra complexity when both spots are both activated, which was not considered in the latter case. While the percentage seems to be low, it is an underestimation of the power of the proposed algorithm because of the regular-density, unoptimized DOT grid. In addition, the parameters in the reconstruction algorithm, and the structural assumptions made on the covariance matrices, can also be optimized for individual cases to improve the accuracy of the spatial priors. Further, the potential EEG distinguishability issues discussed in Sec. 3.3 can also limit the amount of improvement observed. In practice, the DOT optodes would best be optimally placed according to the region of interest, or on should use a large number of optodes for an accurate spatial prior.

To show the improvement using a higher-density DOT, we ran the same simulations using a high-density system that is similar to White and Culver.^21^ The percentages of improved single-spot spatial-only reconstruction and spatiotemporal reconstruction were increased to 92.9% and 69.8%, respectively. The average BSM values in the spatial-only reconstruction without and with DOT prior was 23.11±3.41  mm and 12.44±5.88  mm. When assessing the improvement double-spot reconstruction, the number of combinations where both spots were detected without and with DOT prior were 484 and 410 out of 500 (96.8% and 82.0%), respectively, and among these combinations, the average BSM values at the two locations without and with DOT prior were 28.66±5.55  mm and 25.03±4.72  mm in comparison to 10.62±7.03  mm and 10.93±6.57  mm. While it is expected that the average BSM is further improved due to the higher-density grid and hence more accurate spatial prior, interestingly, the number of combinations where one source was undetected did not decrease. This further indicates the importance of optimizing the DOT grid and individualized choice of reconstruction parameters. See Appendix for details and Sec. 4 for further discussion.

## 4 Discussion

We have proposed a spatiotemporal reconstruction algorithm, which utilizes DOT reconstruction as the spatial prior of EEG reconstruction. We showed that using the proposed algorithm, high spatiotemporal resolution reconstruction of neuronal sources can be achieved, and discussed how to address the potential issues due to the different locations of neuronal sources in respect to electrode or optode locations.

We have shown that in comparison to the DOT-based prior, the projection prior, as was used in Aihara et al.,^19^ has less improvement on the spatial resolution of EEG reconstruction. Another limitation of using a projection method is that it is only applicable for activation on the cortical surface and even there, not inside a sulcus. If activation voxels are also allowed to be deep inside of the brain (rather than only the surface layer, as is in this paper), when constructing the forward model, DOT reconstruction can potentially provide spatial priors for deeper sources as well,^15^ which is not possible for the projection method by definition.^19^ This deserves further study.

When the neuronal sources are both close to one electrode, increasing the number of electrodes and using DOT prior can indeed improve the accuracy of reconstruction, but such improvement can be fundamentally limited as can be seen in the first column of Fig. 9 and Table 2. What is the maximum improvement one can achieve in each case remains to be further studied in future work. Based on the results reported in this paper (Figs. 4–13, Tables 1–3), high spatiotemporal resolution is achievable, where EEG provides the temporal resolution and the basic spatial distinguishability, and DOT provides the spatial accuracy. To be specific, DOT-based prior can only improve the spatial precision when the two activation spots are distinguishable (albeit not necessarily accurately) in EEG. When the spatial information is completely lost in EEG reconstruction, such prior does not enable good spatiotemporal reconstruction, as seen in Fig. 8.

Our results, seen in Fig. 11, suggest that an incorrect prior can lead to worsened reconstruction results. The algorithm can be improved such that it is less prone to the influence of incorrect priors. For instance, the Twomey algorithm^14^ has been shown to be able to effectively handle fMRI-invisible sources in EEG-fMRI studies. In addition, a more carefully chosen decomposition of the covariance matrices ($C_{N}$ and $C_{P}$) can also help improve the reconstruction accuracy.^29^

Apart from improving the reconstruction algorithms, it is also important to study the problem of optimally placing the optodes, because it is a known difficulty in DOT reconstruction that the results heavily depend on the placement of probes, and this problem has been well-studied.^21^^,^^36^^,^^37^ To address this issue, multiple methods have been proposed to optimize the configuration of the DOT grid such that a given region of interest can be best imaged.^37^^–^^39^ In practice, it would be beneficial to utilize such methods to maximize the accuracy of the DOT prior, especially when a high sensor-count system is not available. To further ensure that the two different modalities DOT and EEG are sensitive to the same regions, correspondence of the sensitivities of them can also be assessed.^40^ In future work, the optimal arrangement of optodes will be considered to improve the performance of the proposed algorithm.

In recent studies, especially those focusing on high-density DOT, it is more common to use high-resolution brain meshes with >500,000 nodes.^5^^,^^41^^,^^42^ In this paper, the number of nodes in the brain mesh (∼100,000) is in comparison lower, and may limit the resolution of the reconstruction results due to the limited resolution of the model. In particular, the spread of the reconstruction results can be larger than on finer meshes, because the tetrahedrons are also larger in size. Although the density used here is commonly used in lower-density DOT literature,^15^^,^^40^ we also tested the proposed algorithm on a higher-resolution brain mesh with 660,898 nodes, and demonstrated a similar improvement in the case shown in Fig. 4 (see details in Appendix). The low-resolution mesh is therefore used for lower computational cost. While the resolution of the mesh is sufficient for demonstrating the feasibility of the algorithm, in future work, we will consider using a higher density mesh for potentially higher reconstruction accuracy.

When simulating the DOT data, two important linearization approximations were made: the linear forward model of the DOT system (Sec. 2.1.3) and that hemodynamics are related to neuronal activation via convolution (Sec. 2.3.1). Linearizing the forward model in this fashion is extremely common in the DOT literature.^4^^,^^5^^,^^21^^,^^43^ It is based on the assumption that the fluctuations of the optical properties in the tissue are small during the period of measurement,^21^ which is often true for functional activation. While the hemodynamic response can be non-linear,^44^^,^^45^ in simulation studies, it is common to make the simplifying assumption that hemodynamics can be stimulated by convolving the neuronal activation with a fixed HRF.^2^^,^^30^ In practice, while often a good assumption, linearization here can be a poor approximation in some cases, e.g., in the most extreme case, neuronal and vascular activities can be unsynchronized.^46^ Such violations of the assumption can limit the efficacy of the proposed algorithm and more care should be taken when processing real-life recordings. For example, one can modify the weight of the DOT prior on EEG (the parameters in Sec. 2.3.2), or utilize more robust algorithms such as Twomey,^14^ as discussed already.

Both our proposed method and the work by Aihara et al.^19^ are based on hierarchical Bayesian models. In addition to the differences mentioned in Sec. 1, most fundamentally, our method solves the model using ReML, while in Aihara et al., variational Bayes was used.^19^ Despite the similarity between ReML and variational Bayes,^47^^,^^48^ a big advantage of ReML is that one can easily make structural assumptions on both sensor noise and brain activity covariance matrices, which are essential in DOT reconstruction,^29^ and can also be useful in model selection problems.^47^ In comparison, although assumptions can also be made on the covariance matrices in variational Bayes, this can involve very complicated derivations.^20^

## 5 Appendix

**Fig. 15:**
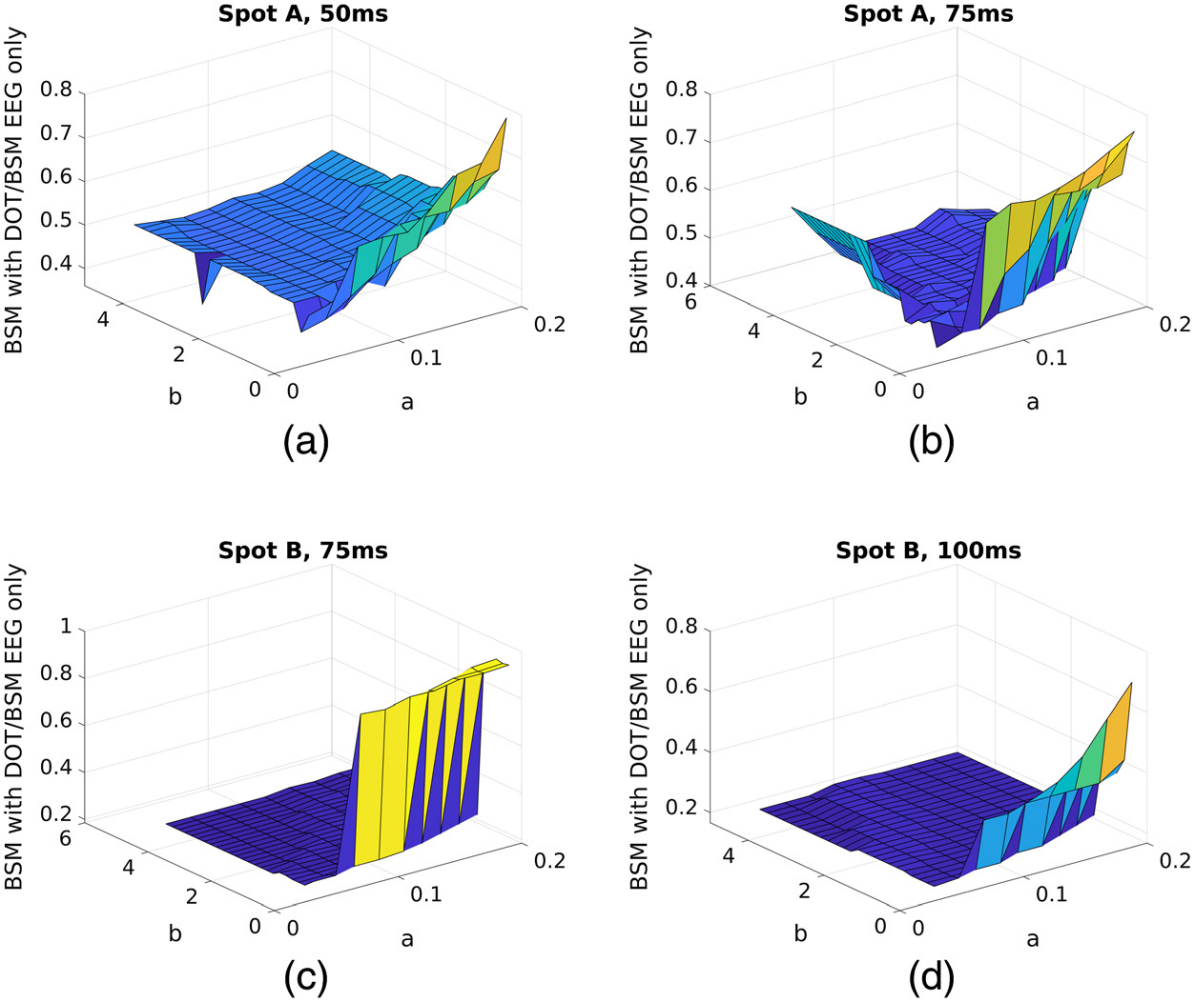
Improvement of BSM values of activation spots A and B at different time points. The grid configuration and locations of the activation spots are the same as in Sec. 3.1. (a) Spot A at 50 ms; (b) Spot A at 75 ms; (c) Spot B at 75 ms; (d) Spot B at 100 ms. It can be seen that in the ranges of approximately a∈(0.05,0.1), b∈(1,5), the exact values of a and b have little impact on the results.

To find the optimal values of parameters a and b in Sec. 2.3.2, we swept a from 0.04 to 0.5 with a step size of 0.02, and b from 0.4 to 5 with a step size of 0.2, and tested the improvement of BSM values (represented by the ratio of BSM with DOT prior to BSM of EEG only) using the case in Sec. 3.1. It can be seen in Fig. 15 that as long as a and b are within a certain range, the results are not very sensitive to the specific values chosen.

**Fig. 16:**
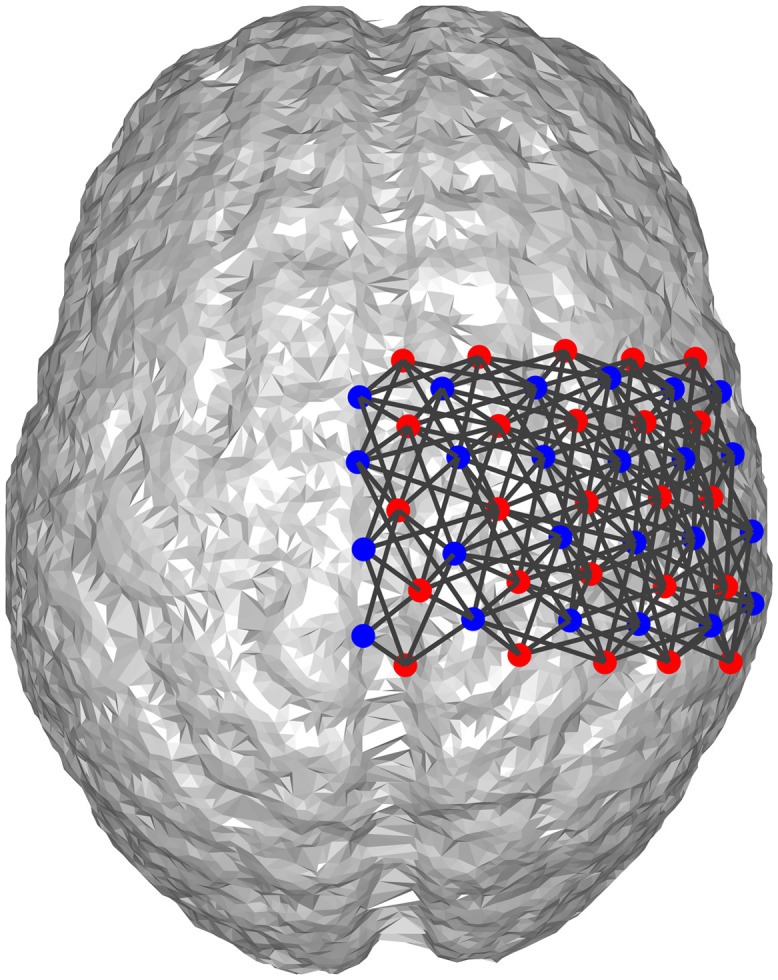
Illustration of the high-density DOT grid. Blue dots: DOT detectors; red dots: DOT sources; black lines: DOT channels formed by source-detector pairs.

**Fig. 17:**
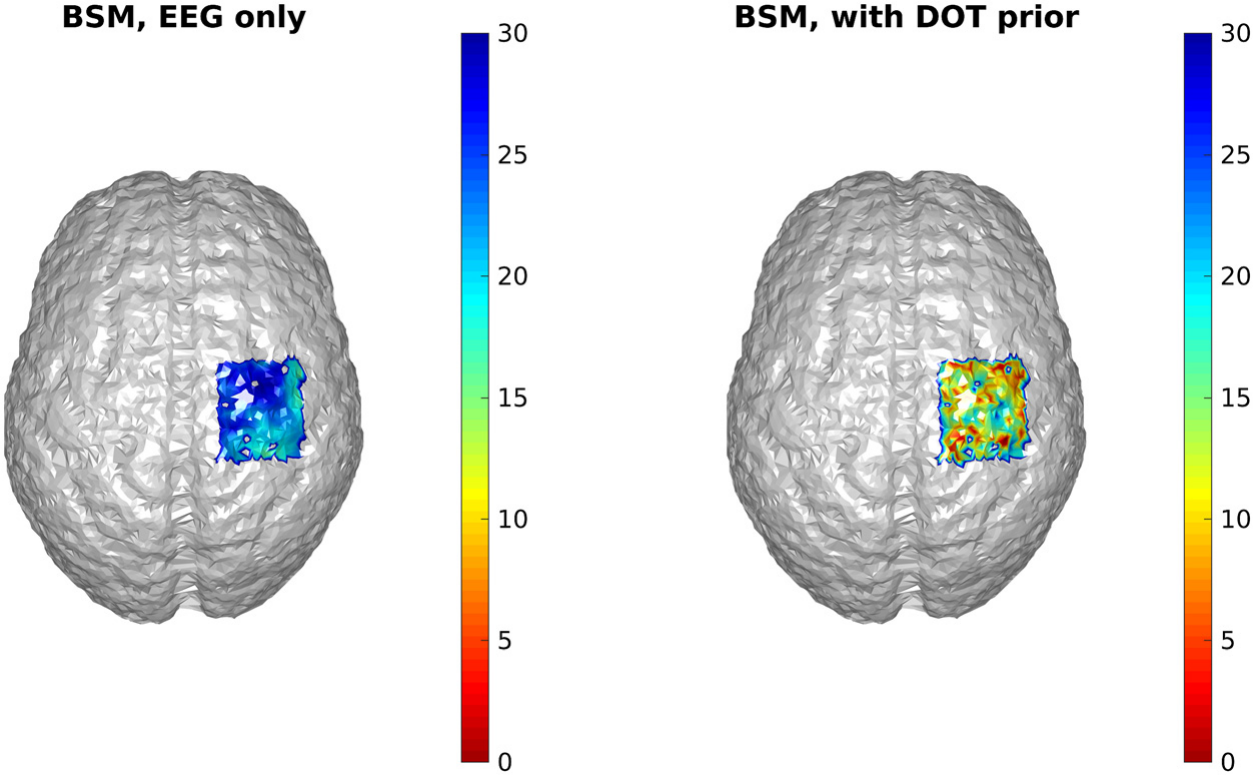
Comparison of BSM values using only EEG and EEG with high-density DOT prior. Shown are 1000 simulated locations, and an overall improvement of reconstruction accuracy as measured by BSM can be observed. It can be seen in comparison to Fig. 14 that the improvement using a high-density DOT grid is more substantial. The colorbars have the units of mm.

To verify that a high-density DOT grid that can more reliably provide the spatial prior can increase the efficacy of the proposed algorithm, we ran the same simulations in Sec. 3.5 using a high-density DOT grid shown in Fig. 16, where the source-detector distances range from 0.75 to 2.9 cm. Using the same standard 32-channel EEG and the same simulation procedure in Sec. 3.5, the improvement of BSM values using DOT prior is shown in Fig. 17, which shows more substantial improvements in comparison to the results in Fig. 14.

To verify that the resolution of the mesh used in the paper suffices to demonstrate the efficacy of the proposed algorithm, we simulated the same case as shown in Fig. 4 using a high-resolution brain mesh. The mesh was generated using the same method described in Sec. 2.1.1, yielding 660,898 nodes, 3,380,723 tetrahedrons, and 93,879 brain source locations. The joint reconstruction pipeline was identical to that described in Sec. 2.3.2, except that spatial smoothness assumption was made when performing DOT reconstruction to reduce spikiness in the results. In particular, instead of the i.i.d. assumptions on ΔHbO and ΔHb, we replaced the identity matrices in $C_{P}$ with G, where $G_{i,j}=\exp({\left(r_{i}-r_{j}\right)}^{2}/\sigma^{2})$, $r_{i}$ is the location vector of the i’th voxel, and σ is the smoothing factor which was chosen to be 2.5 mm.

**Fig. 18:**
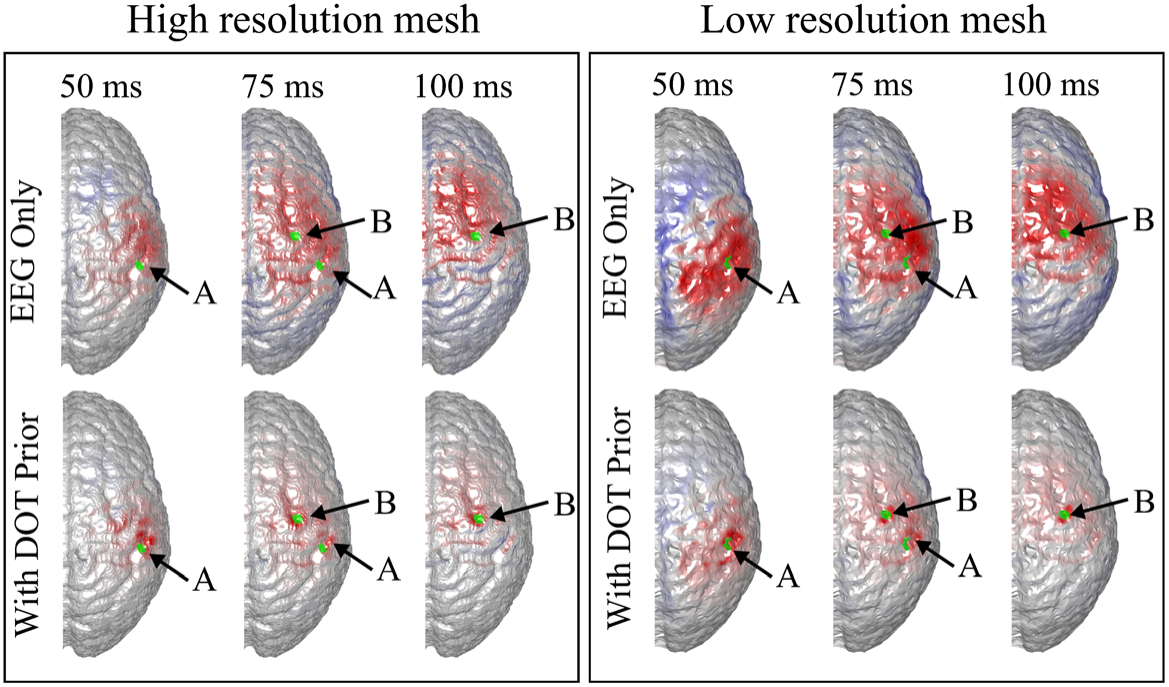
Comparison of true neuronal activation, reconstruction using only EEG, and reconstruction of EEG using DOT prior at different times. The right panel is identical to Fig. 4, and in the left panel, the activation locations are the same as those in Fig. 4, but a high-resolution mesh was used. A similar improvement can be observed. The overlaid green regions indicate the true neuronal activation. Only the right hemisphere is shown, as no significant activation is observed on the left hemisphere.

As is shown in Fig. 18 and Table 4, the spatial accuracy of reconstruction is substantially improved in a similar fashion on both high-resolution and low-resolution meshes, despite small differences. This suggests that while high-resolution brain meshes are desirable for high reconstruction accuracy, the resolution used in this paper is sufficient for our purposes.

**Table 4:** Quantitative comparison of reconstruction using only EEG and EEG with DOT reconstruction prior in the case shown in Fig. 4, but with a high-resolution brain mesh. The BSM values suggest substantial improvements that are similar to those in Table 1.

	50 ms, spot A	75 ms, spot A	75 ms, spot B	100 ms, spot B
EEG only	19.4 mm	31.3 mm	24.3 mm	30.9 mm
With DOT prior	12.4 mm	8.5 mm	5.6 mm	7.7 mm
